# Adaptive robot training for the treatment of incoordination in Multiple Sclerosis

**DOI:** 10.1186/1743-0003-7-37

**Published:** 2010-07-29

**Authors:** Elena Vergaro, Valentina Squeri, Giampaolo Brichetto, Maura Casadio, Pietro Morasso, Claudio Solaro, Vittorio Sanguineti

**Affiliations:** 1 University of Genoa, Department of Informatics, Systems and Telecommunications, Via Opera Pia 13, Genoa, Italy; 2 Italian Institute of Technology, Via Morego 30, Genoa, Italy; 3 Department of Neuroscience, Ophthalmology and Genetics, University of Genoa, Via A. De Toni 5, Genoa, Italy; 4 Department of Neurology, ASL3 Genovese, Genoa, Italy

## Abstract

**Background:**

Cerebellar symptoms are extremely disabling and are common in Multiple Sclerosis (MS) subjects. In this feasibility study, we developed and tested a robot therapy protocol, aimed at the rehabilitation of incoordination in MS subjects.

**Methods:**

Eight subjects with clinically defined MS performed planar reaching movements while grasping the handle of a robotic manipulandum, which generated forces that either reduced (error-reducing, ER) or enhanced (error-enhancing, EE) the curvature of their movements, assessed at the beginning of each session. The protocol was designed to adapt to the individual subjects' impairments, as well as to improvements between sessions (if any). Each subject went through a total of eight training sessions. To compare the effect of the two variants of the training protocol (ER and EE), we used a cross-over design consisting of two blocks of sessions (four ER and four EE; 2 sessions/week), separated by a 2-weeks rest period. The order of application of ER and EE exercises was randomized across subjects. The primary outcome measure was the modification of the Nine Hole Peg Test (NHPT) score. Other clinical scales and movement kinematics were taken as secondary outcomes.

**Results:**

Most subjects revealed a preserved ability to adapt to the robot-generated forces. No significant differences were observed in EE and ER training. However over sessions, subjects exhibited an average 24% decrease in their NHPT score. The other clinical scales showed small improvements for at least some of the subjects. After training, movements became smoother, and their curvature decreased significantly over sessions.

**Conclusions:**

The results point to an improved coordination over sessions and suggest a potential benefit of a short-term, customized, and adaptive robot therapy for MS subjects.

## Background

Multiple Sclerosis (MS) is associated with a variety of symptoms and functional deficits, in proportions that change widely from patient to patient. About 30% of subjects show functionally relevant cerebellar deficits [1]. The most common symptoms are tremor [2,3] and ataxia [4]. Cognitive deficits have been reported as well [5]. Ataxia in particular implies an inability to perform coordinated movements that involve multiple joints [6]. In these subjects, movements typically result in curved trajectories and prolonged durations. All these symptoms are highly disabling and resistant to treatment.

Even though evidence for efficacy of rehabilitation came from studies with subjects with chronic progressive MS [7], there is growing evidence that subjects with relapsing-remitting MS may benefit from rehabilitation interventions [8]. Recent reviews suggest that exercise therapy can be beneficial for subjects with MS [9] and that multi-disciplinary rehabilitation programs may improve their experience in terms of activity and participation, but cannot change the level of impairment [10]. Due to the different degrees of impairments in different MS subjects, it is crucial that in these subjects the timing and mode of rehabilitation treatment are set individually.

As regards cerebellar symptoms in MS subjects, there is no conclusive evidence on the efficacy of neuro-rehabilitation treatments [11]. Physiotherapy approaches have resulted in small, short-term improvements in gait [12], balance [13,14] and arm [13] functions. Repetitive transcranial magnetic stimulation (rTMS) on the motor cortex has been reported [15] to induce a short-term improvement in coordination. Cooling of the limbs was reported to decrease tremor, but not incoordination [16,17].

Robot therapy has been shown effective in promoting the recovery of stroke subjects [18]. It is natural to wonder if it can be of any use in MS subjects, in particular those with cerebellar symptoms. Very few studies have addressed the application of robot-assisted treatments to MS subjects, targeting gait [19,20] and movements of the upper limb [21].

A prerequisite for rehabilitation, either robot- or therapist-assisted, is that subjects preserve their ability to adapt to novel dynamic environments [22]. Recent studies have demonstrated that MS subjects with no disability have a preserved capability of predicting the effects of robot-generated forces [23]. Moreover, MS subjects with severe impairment have at least a residual capability for sensorimotor adaptation in arm [24] and posture [25] control.

Cerebellar deficits have been associated with an inability to adapt to novel dynamic environments [26,27]. These subjects may possibly benefit from adaptive training protocols [28], in which robots do not just assist subjects while they practice movements but, rather, they provide unfamiliar dynamic environments to which subjects are required to adapt. These approaches have been investigated in the rehabilitation of chronic stroke survivors [29]: improvement is greater when robot-generated forces are directed toward magnifying the original movement errors (i.e. lateral deviation), with respect to situations in which forces tend to reduce (and possibly reverse) such errors.

In this study, we investigate a robotic approach to neuro-motor rehabilitation of MS subjects that combines, in the same protocol, the evaluation of motor performance and the fine tuning of the training exercise. More specifically, we developed a personalized adaptive training protocol, where subjects are required to adapt to dynamic environments that either enhance or oppose (i.e., reduce or even reverse) the motor errors which result from impaired coordination.

We specifically asked (i) which approach (error-enhancing, error-reducing) would be more effective and, more in general, (ii) whether robot therapy - more specifically, adaptive training - could be beneficial to cerebellar MS subjects.

## Methods

### Subjects

Eight subjects with clinically definite MS according to McDonald criteria [30] participated in this study (3 M + 5 F, age 48 ± 14 - mean ± SD).

Inclusion criteria were both sexes, age older than 18 years, stable phase of the disease, without relapses or a worsening greater than 1 point at the Expanded Disability Status Scale (EDSS) [31] score in the last three months and with an EDSS lower than 7.5, presence of cerebellar signs such as kinetic/intention tremor and incoordination at the upper limb. In order to have subjects with prevalent cerebellar deficits, we selected subjects with Scripps' Neurological Rating Scale (NRS) [32] scores for the upper extremity (0: severe, 1: moderate, 3: mild, 5: normal) greater or equal to 3 (mild) for sensory and motor system deficits, and lower or equal to 3 (mild) for cerebellar deficits.

The exclusion criteria were previous utilization of robot-therapy, spasticity (Ashworth scale score greater than 1 evaluated at the elbow and shoulder), presence of nystagmus, visual acuity less than 4 (out of 10), kidney or liver disease and pregnancy; relapses within the last three months, treatment with corticosteroids within the previous three months, use of anti-epileptic drugs, benzodiazepine, antidepressants, β-blockers, drugs for spasticity initiated within the last two weeks, Mini-Mental State Examination (MMSE) < 24.

Disease duration was 11 ± 6 years. Disability - quantified by the EDSS - was 5 ± 1. The degree of impairment of the motor, sensory and cerebellar systems, as it relates to upper limb function, was assessed through the 'arm' portion of the Scripps' NRS, separately for the two arms. The same neurologist examined all the subjects. Detailed demographic information is reported in Table 1.

**Table 1:** Clinical data for the experimental subjects.

Subject	Age (y)	Sex	Hand	Disease Duration (y)	Disease Course	EDSS (0-10)	MODE
S1	38	M	R	14	RR	6.5	EE+ER

S2	41	F	L	15	SP	3	EE+ER

S3	61	F	R	3	SP	4	ER+EE

S4	42	F	R	8	RR	4.5	ER+EE

S5	73	M	L	4	SP	4.5	EE+ER

S6	34	F	L	11	SP	5	ER+EE

S7	59	M	R	20	SP	6.5	EE+ER

S8*	37	F	R	4	SP	6	ER+EE

Total	48 ± 14			10 ± 6		5 ± 1	

RR: relapsing-remitting; SP: secondary-progressive. Subject 8 dropped out the study.

The research conforms to the ethical standards laid down in the 1964 Declaration of Helsinki that protects research subjects and was approved by the competent Ethical Commitee. Each subject signed a consent form that conforms to these guidelines.

### Task

Subjects sat on a chair, with their torso and wrist restrained by means of suitable holders, and grasped the handle of a planar robotic manipulandum [33] with their most affected hand. The position of the seat was also adjusted in such a way that, with the cursor pointing at the center of the workspace, the elbow and the shoulder joints were flexed about 90° and 45°, respectively.

We used an adaptive training paradigm, which was previously shown effective in stroke subjects [28,29,34]. The task consisted of reaching movements in three different directions, starting from the same center position. The targets were presented on a 19" LCD computer screen, placed in front of the subjects, about 1 m away, at eye level. Targets were displayed as round green circles (diameter 1 cm) against a black background. The current position of the hand was also continuously displayed, as a yellow circle (diameter 0.5 cm). The nominal amplitude of the movements (distance of the targets from the center position) was 10 cm. The sequence of target presentations alternated the central target and one of the three peripheral targets (directions 30°, 150°, 270°), generated in random order.

To decrease movement variability, subjects were encouraged to keep an approximately constant timing. As reaching movements are characterized by a bell-shaped velocity profile [35], for each movement we estimated the peak value of hand speed, and provided a feedback/reward to the subject if this value was comprised within the 0.25-0.55 m/s range, which corresponds to a movement duration of 0.7-1.5 s. If the measured speed was smaller or greater than the above range, the colour of the target was changed to white or red, respectively.

The experiment was organized into epochs, each consisting of the presentation of all three targets (one for each direction), in random order. Each rehabilitation session consisted of six phases:

(i) Familiarization (5 epochs, i.e. 15 movements). Subjects became familiar with the manipulandum - which did not generate forces - and with the task;

(ii) Baseline 1 (5 epochs, i.e. 15 movements). The robot did not generate forces. For each target, we identified the subject's 'average' trajectory, as the mean of all five trajectories toward that target.

(iii) Robot Training (40 epochs, i.e. 120 movements). By means of an iterative procedure (see below) the robot learned the forces necessary to generate lateral perturbations (forces directed orthogonally with respect to the trajectory) that, for each target direction, either enhanced or decreased (and possibly reverse) the lateral deviation of the 'average' trajectories estimated during the Baseline 1 phase (error-enhancing, EE, or error-reducing sessions, ER, see below). To prevent subject adaptation, the robot only generated forces in 1/4 of the movements (selected randomly).

(iv) Baseline 2 (5 epochs, i.e. 15 movements). A second unperturbed baseline phase, aimed at checking whether the baseline pattern had changed.

(v) Subject training (96 epochs, i.e. 288 movements). Subjects were continuously exposed to the forces that the robot had previously learned (force trials, i.e. movements where force is turned on) with no more adjustments. To monitor the progress of adaptation, in the last portion of this phase (last 56 epochs), in 1/8 of the movements the force was unexpectedly removed (catch trials). This fraction of catch trials on the total of movements was chosen to provide enough information to allow statistical analysis while avoiding, at the same time, that adaptation occurs more slowly because of the perceived uncertainty in the dynamic environment [36].

(vi) Wash-out (15 epochs, i.e. 45 movements). Forces were turned off to assess the persistence of the induced adaptation (if any).

**Figure 1:**
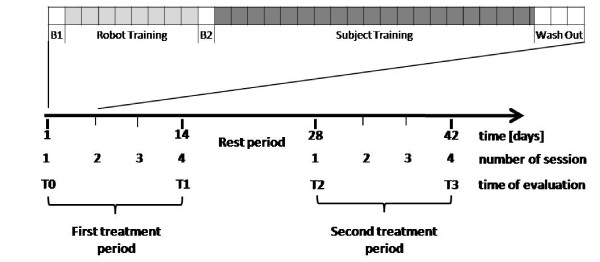
**Training protocol and study design**. Top: Phases of the training protocol: Baseline 1 (B1), Robot Training, Baseline 2 (B2), Subject Training, Wash-out. The phases in which the robot generates no forces (B1, B2, Wash-out) are indicated in white. Each square corresponds to five epochs. Bottom: Overall study design, indicating the treatment and rest periods and the times of evaluation (T0-T4).

Therefore, a complete session included 166 epochs (i.e. 498 movements), and lasted approximately 60 minutes. Figure 1 (top) summarizes a schematic description of the training protocol.

### Robot Training procedure

**Figure 2:**
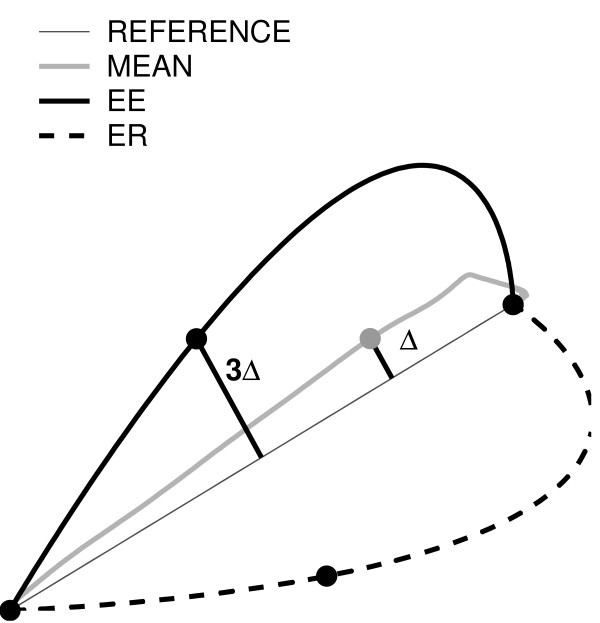
**Desired trajectory construction**. Maximum lateral deviation (Δ) from the nominal path calculated after the evaluation of the mean trajectory (grey). It is tripled (3Δ) and centered. The corresponding point became the via-point for minimum-jerk trajectory that enhance (black line) or reduce (black dotted line) subject's error.

An iterative algorithm, similar to that proposed in [28], was used to estimate and store the time profile of the forces, to be generated by the robot during the subsequent Subject Training phase. The algorithm aims at determining the forces that shift a subject's trajectory toward a 'reference' trajectory, *x*^D^*(t)*. The 'reference' trajectory, *x*^D^*(t)*, was defined as a 'minimum jerk' trajectory passing through three points [37]: the center, the target and a third via-point; see Figure 2.

We defined the via-point, placed at half the distance from the starting point to the target, and shifted it laterally, of three times the maximum lateral deviation observed in the average baseline trajectory. The 'average' trajectory was the 'average' of all trajectories in the same direction during the Baseline 1.

In error-enhancing (EE) sessions, the shift was on the same side as the lateral deviation observed in the average trajectory. In error-reducing (ER) sessions, the shift was on the opposite side.

The force generated by the robot in direction d = 1...3, F_d_(t), was only present during the initial 2/3 of the total duration of the movement (estimated from that of the 'average' trajectory). This is because we were interested in affecting the early portion of the movements, which best reflects the operation of the feed-forward component of control. Late portions of the trajectory are highly variable, as they reflect the feedback corrections that are likely due to errors in the early portion.

We initially set  for each t, and subsequent movement repetitions were used to adjust the force according to the following update rule [28], where d is target direction (d = 1..3):(1)

The parameter *μ *is a learning rate, which was been heuristically set in the range of 10-30 N/m. If *μ *is too large, the robot training procedure becomes unstable, if *μ *is too small convergence would take too long. In all experiments, we used *μ *= 30 N/m.

As a consequence of this procedure, in EE sessions, forces led to enhancing the lateral deviation of the baseline trajectory. In contrast, in ER sessions, forces opposed - reduced, and ultimately reversed - the initial lateral deviation. For safety reasons, the forces generated by the robot were limited to the ± 14 N range.

### Study design

The rehabilitation protocol included a total of 8 sessions. To compare the two variants of the robot therapy treatment, we used a randomized double blind crossover design. In four consecutive sessions (2 sessions/week), subjects were trained with one type of error-enhancing (EE) forces. In the remaining four sessions (2 sessions/week), forces were error-reducing (ER). The order of application of the two treatments was randomized over subjects - four subjects started with EE training, four subjects started with ER training. The two treatment periods were separated by a 2-weeks rest period.

Figure 1 (bottom) summarizes the study design.

Note that the forces used for training were calculated at the beginning of each session. Therefore, the protocol automatically adapted to the patient's specific impairment, as well as to the improvements - if any - that occurred from session to session.

Subjects were blind with respect to the training protocol, in the sense that they did not receive a detailed explanation of the modalities of generation of force by the robot. Moreover, each subject had peculiar patterns of incoordination and the applied forces were highly direction-specific. Therefore, it is unlikely that they could distinguish among either modality and that they saw forces as something different than mere perturbations.

Clinical testing included the evaluation of the following clinical scales: EDSS and Functional Systems Score [31], Scripps' NRS [32], Ashworth scale [38], the Ataxia and Tremor scales [39], the Nine-Hole Peg Test (NHPT) [40], a Visual Analog Scale (VAS) for upper limb tremor (0-10 score), a self-administered Tremor in Activity of Daily Life (TADL) questionnaire [41]. Subjects and the evaluating clinician were blind with respect to the training protocol (ER or EE).

We made a total of four assessments, at T0 (baseline-day 1), T1 (after 4 sessions - day 14), T2 (after the rest period - day 28) and T3 (after 8 sessions - day 42).

We looked at both specific differences in the two treatments and at the overall effect of robot treatment over the whole duration of the trial.

### Data Analysis

Hand trajectories were sampled at 100 Hz. The *x *and *y *components were smoothed with a 4^th ^order Savitzky-Golay filter (window size 200 ms, equivalent cut-off frequency 6.6 Hz), which was also used to estimate the first three time derivatives. We then estimated the following indicators:

- Lateral deviation of hand trajectory (root mean square value).

- Movement duration, i.e. time elapsed between movement onset and termination; movement onset was identified as the first time instant when hand speed exceeds a threshold (20% of peak speed); movement termination was computed as the first time instant after onset in which movement speed goes below the threshold.

- Symmetry: ratio between the durations of acceleration and deceleration phases.

- Jerk (Teulings') index: root mean square of the jerk (third time derivative of the trajectory), normalized with respect to movement amplitude and duration [42].

Lateral deviation was also used to assess the subjects' ability to adapt to the force patterns provided by the robot.

### Outcome measures

As a primary outcome measure, we took the change in the Nine Hole Peg Test (NHPT) [40], a quantitative scale for distal upper limb function (the test involves the subject placing 9 dowels in 9 holes. Subjects are scored on the amount of time it takes to place and remove all 9 pegs). The test was preceded by a familiarization phase to extinguish learning effects. We took a 20% decrease as the threshold for clinical significance [43,44]. Kinematic (jerk index, lateral deviation, movement duration and symmetry of the speed profile) and clinical indicators (Scripps' NRS, Ataxia score, VAS for upper limb tremor, TADL) were taken as secondary outcome measures.

### Statistical analysis

To compare the effects of the two treatments (EE and ER), to account for the crossover design we analysed the primary outcome measure by using a mixed-effect model [13], with period (first, between T0 and T1, and second, between T2 and T3) and treatment (EE or ER) as fixed factors, subject as random factor and the baseline value at the start of the relevant period (i.e., T0 and T2) as covariate. This adjustment allows us to reduce the observed variation between the two groups of subjects caused not by the treatment itself but by variation of the clinical scale at the beginning of the therapy.

To test the overall effect of adaptive training, we compared the primary outcome measures (change in the clinical scores) between the baseline (T0) and the end of the treatment (T3), irrespective of the training mode (treatment).

As regards the kinematic indicators, we ran a repeated-measures ANOVA with three factors: session (early vs late, i.e. 1 vs 4), phase (baseline 1, baseline 2, late wash-out - last 5 epochs) and treatment (EE, ER). Significant period and session effects would indicate, respectively, that subjects modify their behaviour within and between sessions. To quantify whether the session effect was indeed an improvement, we also directly compared (planned comparisons) session 1 and session 4, for the two treatments taken together and separately for each training mode. As regards changes within one session, to distinguish between the changes in performance occurring during the Robot Training phase from those occurring during the Subject Training phase, we directly compared (planned comparisons) Baseline 1 and Baseline 2 (effect of Robot Training), Baseline 2 and Wash-out (effect of Subject Training) and finally Baseline 1 and wash-out (overall phase effect).

## Results

Seven subjects successfully completed the protocol. Subjects were allowed to rest between consecutive blocks of trials. However, no one did, and in fact the task was well tolerated. Furthermore, there was no degradation of performance at the end of the adaptation phase as compared to the final portion of the wash-out phase. One subject (S8) did not complete the second half of the trial, for reasons unrelated to the study protocol. This subject was excluded from all subsequent analyses.

**Figure 3:**
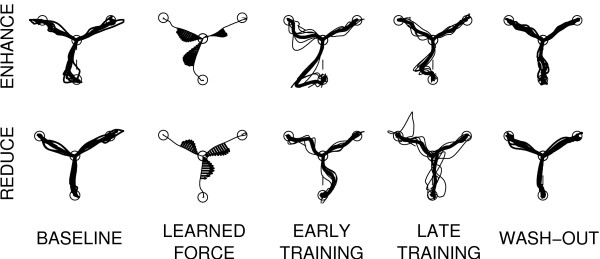
**Typical trajectories**. Typical trajectories during an EE (top) and an ER (bottom) training session. From left to right: baseline trajectories, learned forces, early and late training and late wash-out.

Figure 3 shows typical trajectories from the center position to the three targets, during the different phases of an error-enhancing (top) and an error-reducing session (bottom).

As expected, the forces learned by the robot at the end of the Robot Training phase reflect the average patterns of curvature observed during the baseline phase.

### Primary outcome

We first tested for differences in the training mode. We found a significant effect of period (F(1,6) = 16.004; p = 0.00283). On average, the decrease in the NHPT score was -9 ± 3 s in period 1 and -1 ± 3 s in period 2. However, we found no significant treatment and baseline effects. On average, the NHPT score decrease was -9 ± 5 s in period 1 of error-enhancing sessions, and -9 ± 5 s in the same period of error-reducing sessions.

These results indicate that most of the improvement occurs in period 1, irrespective of treatment type and baseline value.

**Figure 4:**
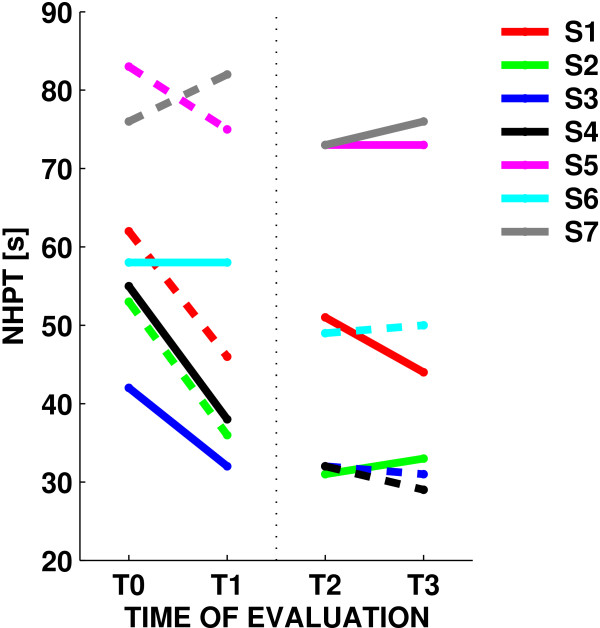
**Nine Hole Peg Test**. Changes in the Nine-Hole Peg Test score for the seven subjects, during error-enhancing (dashed lines) and error-reducing trials (solid lines).

We then looked at the NHPT change from baseline (T0) to the end of the treatment (T3), irrespective of the training mode. In this case, the NHPT score decreased from 61 ± 14 s to 48 ± 20 s, a 24% change (F(1,6) = 16.495, p = 0.007); see also Figure 4. In four subjects, the improvement was greater than 20% (the threshold for clinical significance). One subject displayed a 47% change; no subjects showed significant worsening.

During the first four sessions, irrespective of the training mode, the average score decreased (F(1,6) = 6.7955, p = 0.04021) from 61 ± 14 s to 52 ± 20 s (a 21% change). A smaller decrease, from 49 ± 18 s to 48 ± 20 s (a 4% change) was observed during the last four sessions. Although these results suggest a plateau effect for the improvement in the NHPT score, subjects who improved during period 1 exhibited an additional improvement in period 2 (correlation between changes in the two periods: 0.61); see Table 2.

**Table 2:** Changes in NHPT.

		**NHPT [s]**				**NHPT change [s]**		
			
**Subject**	**Sequence**	**T0**	**T1**	**T2**	**T3**	**Period 1 (T1-T0)**	**Period 2 (T3-T2)**	**Overall (T3-T0)**
	
S1	EE+ER	62	46	51	44	-16	-7	-18
S2	EE+ER	53	36	31	33	-17	2	-20
S3	ER+EE	42	32	32	31	-10	-1	-11
S4	ER+EE	55	38	32	29	-17	-3	-26
S5	EE+ER	83	75	73	73	-8	0	-10
S6	ER+EE	58	58	49	50	0	1	-8
S7	EE+ER	76	82	73	76	6	3	0
S8*	ER+EE	57	61	NA	NA	4	NA	NA
	
Total		**61 ± 14**	**52 ± 20**	**49 ± 19**	**48 ± 20**	**-9 ± 9**	**-1 ± 3**	**-13 ± 9**

### Secondary outcome: clinical scales

The Ataxia score decreased from T0 and T3, irrespective of the training mode (F(1,6) = 6.1935, p = 0.04725). The decrease occurred during the first four sessions (F(1,6) = 10.500, p = 0.01768); no further decrease was found in the late sessions. As regards tremor, the TADL score decreased in the first four sessions, but only with EE training (F(1,6) = 14.087, p = 0.00947); see Table 3. Other clinical scales showed small improvements for at least some of the subjects, but no significant effects were observed.

**Table 3:** Changes in clinical scales.

Subject		Scripps' NRS (5-0)			Ataxia (0-8)	TADL (25-100)	VAS tremor (0-10)		Scripps' NRS (5-0)			Ataxia (0-8)	TADL (25-100)	VAS tremor (0-10)
										
		M	S	C					M	S	C			
S1	**T0**	5	5	1	5	37	5	**T2**	5	5	1	3	35	4.5
	**T1**	5	5	3	3	32	3.5	**T3**	5	5	1	3	35	4

S2	**T0**	3	3	1	5	42	5	**T2**	3	3	3	3	40	4
	**T1**	3	3	3	3	40	4	**T3**	3	5	3	3	40	3

S3	**T0**	3	5	3	3	45	5	**T2**	5	5	3	2	45	5
	**T1**	5	5	3	2	45	5	**T3**	5	5	3	2	45	5

S4	**T0**	3	3	1	5	63	5	**T2**	5	3	1	3	62	4
	**T1**	5	3	1	4	62	5	**T3**	5	3	3	2	60	4

S5	**T0**	5	3	3	3	47	8	**T2**	5	3	3	3	45	6
	**T1**	5	3	3	3	47	7	**T3**	5	3	3	3	45	6

S6	**T0**	5	3	3	3	51	5	**T2**	5	3	3	3	45	5
	**T1**	5	3	3	3	51	5	**T3**	5	3	3	3	45	5

S7	**T0**	5	5	1	3	44	6	**T2**	5	5	1	3	41	5
	**T1**	5	5	1	2	44	5	**T3**	5	5	1	3	41	5

S8*	**T0**	5	5	1	3	63	9	**T2**	-	-	-	-	-	-
	**T1**	5	5	1	3	71	10	**T3**	-	-	-	-	-	-

MSC: Motor, Sensory and Cerebellar

### Secondary outcome: changes in movement kinematics

**Figure 5:**
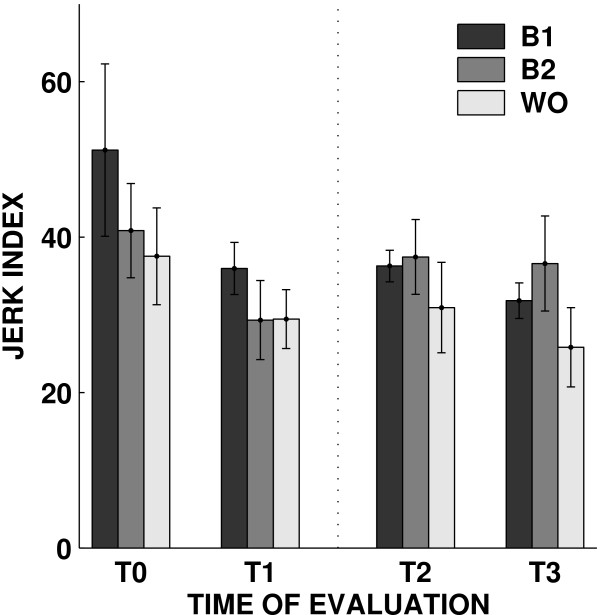
**Jerk index**. Changes in jerk index over sessions. The bars represent the mean value of the indicator over subjects in the baseline1 (B1), baseline2 (B2), wash-out phase (WO).

We found no significant effects of Robot Training (baseline 1 vs baseline 2). As regards the effect of Subject Training (baseline 2 vs wash-out), we found a decrease in the jerk index (F(1,6) = 13.632, p = 0.01018), i.e. after Subject Training movements tend to be smoother - but this same effect was no longer significant when considering baseline 1 vs late wash-out; see Figure 5.

**Figure 6:**
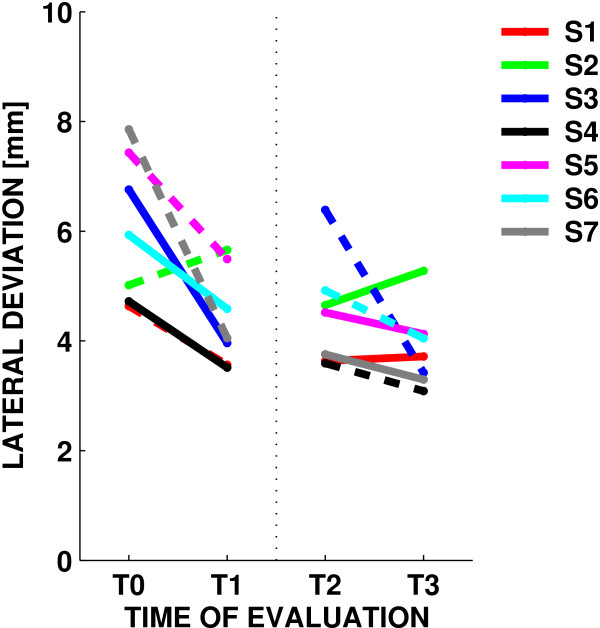
**Lateral deviation**. Changes in lateral deviation over sessions. Dashed lines indicate EE sessions and solid lines refer to ER sessions.

Moreover, we found no significant improvements in movement duration, speed profile symmetry and trajectory curvature (as measured by the lateral deviation). Overall, these results suggest that Subject Training consistently increases movement smoothness, whereas mere exercise - the Robot Training phase - does not have a consistent effect. As regards the effect of session, we found no significant effects for duration, speed profile symmetry or the jerk index. However, we found a significant decrease in trajectory curvature (F(1,6) = 19.801, p = 0.00433); see Figure 6.

### Error-enhancing vs error-reducing training

In all indicators the effect of the training mode (EE vs ER) was not significant except the TADL secondary outcome that significantly decreased only in EE training (F(1,6) = 14.087, p = 0.00947). Likewise, in no indicator we found significant interactions between the training mode and the other factors. Finally, as regards trajectory curvature, we found that most of the decrease occurred during the first block of four sessions, irrespective of the training mode (F(1,6) = 17.767, p = 0.00559, sessions 1 vs 4; and F(1,6) = 8.6312, p = 0.02602, sessions 5 vs 8).

### Force field adaptation

**Figure 7:**
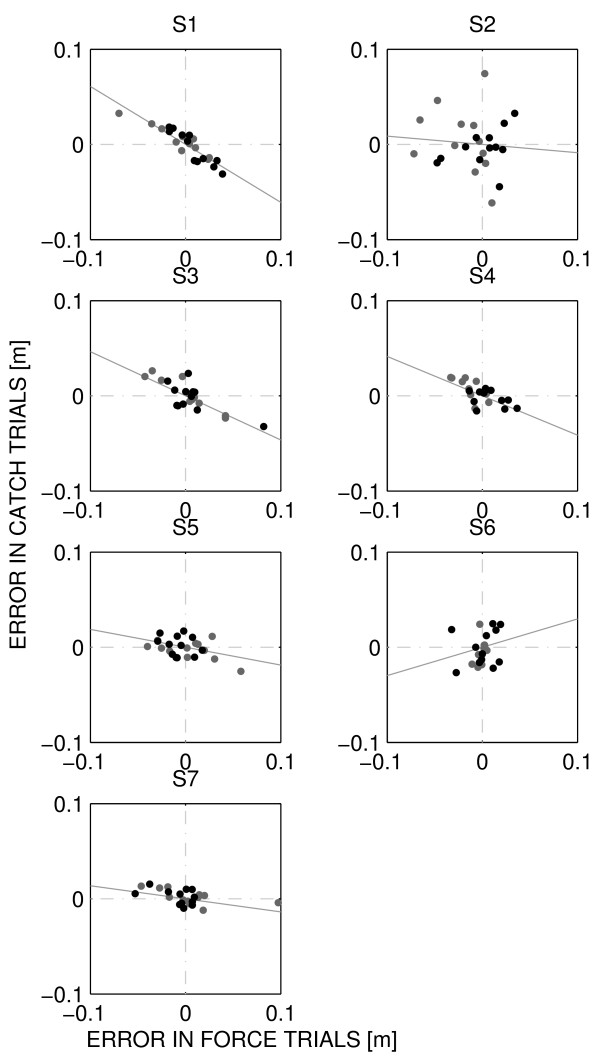
**Motor adaptation by subject**. From top to bottom: subjects 1-7. Grey and black dots indicate ER and EE sessions respectively. The grey line represents the regression line. Adaptation is indicated by the negative correlation between the error in early force trials and that in late catch trial.

To assess the capability of adapting to the forces generated by the robot during the Subject Training phase, we used a 'learning index' [26] that compared some signed measure of execution error (here, maximum lateral deviation) in movements where force is turned on (force trials) and where force is turned off (catch trials). If adaptation had occurred, the execution error observed in early force trials should be negatively correlated with the same motor error, observed in the late catch trials. For each subject we displayed the error in early force trials versus the error in late catch trials. The results, for each subject and for each training mode, are shown in Figure 7.

The slopes of the regression lines can be used to quantify the amount of adaptation. The estimated slopes, as well as the corresponding correlation coefficients r are, -0.61 (S1, r = 0.80), -0.09 (S2, r = 0.01), -0.46 (S3, r = 0.63), -0.41 (S4, r = 0.49), -0.19 (S5, r = 0.18), 0.30 (S6, r = 0.07), -0.14 (S7, r = 0.32). These results suggest that five subjects display signs of adaptation (negative slope, substantial correlation) to the force generated by the robot. Two subjects have small correlation, suggesting that little or no adaptation occurred.

Although the correlation was not significant, subjects displaying a greater NHPT improvement were also those displaying a greater amount of adaptation.

## Discussion

In this feasibility study, we developed an adaptive robot training technique, and applied it to MS subjects with cerebellar symptoms, i.e. ataxia, tremor or both.

### Adaptive robot training improves upper limb function

Across sessions, we found a significant decrease in the NHPT score - a quantitative measure of arm-hand coordination. Additional evidence for improved coordination is provided by the decreases in the ataxia and tremor scores (period 1, EE sessions only). Kinematic analysis of motor performance supports these results. At the end of a training session, movements become significantly smoother. In addition, over sessions, the curvature of movement trajectories decreases significantly.

The improved NHPT score is particularly remarkable, as it suggests that the improved coordination may transfer to tasks more related to activities of daily living [21]. In contrast, robot therapy in stroke subjects displays little generalization to movements that had not been explicitly trained [45,46].

Most subjects showed a clear improvement in the first four sessions, and only few improved further in the second half of the training protocol. However, improvements in the first period predicted an additional (smaller) improvement in the second period. This says little on how many sessions could be appropriate to maximize subjects' benefit, but suggests that improvement in early sessions is predictive of a further improvement.

Is the observed improvement due to the robot, or it is just the effect of repeated exercise? Within a session, improvements were only observed after Subject Training, whereas Robot Training - during which the robot exerts no forces in 75% of the movements - did not appear to have an effect. This observation points to a specific within-session effect of the robot (robot-assisted Subject Training phase) when compared to exercise alone. These short-term effects, as well as adaptive processes that occur at different time scales [47] may contribute to the overall observed (between-session) performance improvements.

### Error-enhancing vs error-reducing training

Previous studies [29,34] on chronic stroke survivors suggested that adaptation to error-enhancing perturbations (error-enhancing training) can induce short-term improvements of motor performance. In contrast, adaptation to perturbations that opposed the initial lateral deviations (error-reducing training) induced a slight worsening of performance [29].

In the present study, we found no significant differences - neither short-term (within session) nor long-term (between sessions) - between error-enhancing and error-reducing training. This may be due at least in part to the small number of sessions and/or subjects. Furthermore, as noted in the Methods, the 'error-reducing' modality may actually tend to augment the error - although in an opposite direction with respect to the initial lateral deviation - so they may be no different in terms of recovery.

Actually, the cited study on stroke subjects only focuses on short-term (one session) effects, and it is unclear what effect would be expected over multiple sessions. Therefore, that study cannot be directly compared to our findings. Nevertheless, the latter may indicate that stroke survivors and MS subjects with cerebellar symptoms have distinctly different modalities of recovery.

In stroke subjects, recovery might be mostly driven by motor errors, so that it would be greater and/or faster if errors are amplified. Little is known about the mechanisms underlying functional recovery (if any) of MS subjects with cerebellar symptoms. However, one possible hypothesis is that in these subjects recovery may be facilitated by exercises that challenge their ability to deal with novel dynamic environments, for which the cerebellum plays an essential role [26,27]. As a consequence, in these subjects recovery may not depend on the specific dynamic environment to which to adapt but, rather, on the mere task of adapting.

Further experiments are needed to test this working hypothesis.

It should be noted that the cross-over study design as a number of limitations. The effect of exercise during the first period does not vanish during the 2-weeks rest period. This is partly accounted for by the statistic procedures (performance at the beginning of treatment periods taken as covariate), but existing differences in the two treatment modalities as well as an interaction among them cannot be completely ruled out. Additional studies would be needed, involving more subjects and two separate treatment groups.

### MS subjects adapt to unfamiliar dynamic environments

In adaptive training, robots do not just assist subjects while they practice movements (or resist to them) but, rather, they provide unfamiliar dynamic environments, to which subjects are required to adapt. Stroke subjects are capable to adapt to these environments, and when the latter are removed, after wash-out ot the after effects they exhibit improved coordination [34]. These studies, together with evidence of reorganization of the motor cortex driven by motor skill learning [48] have suggested that the neural processes associated with implicit motor adaptation may reshape the sensorimotor mappings altered by stroke [49]. The same cortical reorganization occurs in subjects with early MS, and might contribute to limit the consequences of irreversible tissue damage in lesions and normal-appearing brain tissue [50]. This would suggest that rehabilitation of MS subjects should primarily aim at facilitating the emergence/reorganization of compensatory strategies. Adaptive training seems an attractive way to promote such reorganization and, consequently, particularly promising for rehabilitation of MS subjects, who display different types and degrees of deficit, often with an important cerebellar component.

This pilot study provides new evidence that MS subjects are able to adapt their arm movements when they are exposed to a robot-generated force field. More specifically, our results suggest that, when the robot interacts with subjects performing movements, it is capable to achieve a consistent pattern of force to either enhance or reduce the subjects' errors. A comparison of the errors made during the early force trials and those made during the late catch trials clearly demonstrated that MS subjects are capable of adapting to both error-enhancing and error-reducing force fields.

## Conclusions

This study suggests that adaptive-type robot therapy may be a useful and safe approach to improve cerebellar symptoms in MS subjects.

In particular, the finding that six subjects (out of 7) showed a clinically significant improvement in NHPT in pre-post analysis and an improved coordination is specially remarkable, as most medications and rehabilitation approaches are little effective with cerebellar symptoms.

However, unlike stroke subjects, we could not observe a clear difference in the effect of the two treatments (error-enhancing, error-reducing). This may indicate a different modality of recovery of these subjects with respect to stroke survivors. While in stroke subjects recovery is driven by motor errors, in MS cerebellar subjects recovery may be triggered by the mere adaptive training, irrespective of the specific perturbing environment). In fact, in our subjects the overall improvement was associated with a preserved ability, within a session, to adapt to unfamiliar dynamic environments.

We could not conclude on the ideal number and duration of the treatment sessions. However, most of the improvement occurred in the early exercise sessions (period 1) and its magnitude was predictive of additional improvements in later sessions (period 2).

The above conclusions need to be taken cautiously because of the limited size of our sample, and should be confirmed in a larger study. Nevertheless, this study may represent a starting point toward designing novel robot therapy approaches and to expand the range of application of robots in neuromotor rehabilitation.

## Competing interests

The authors declare that they have no competing interests.

## Authors' contributions

The overall design of the experiment was agreed by all authors after extensive discussions. ViS and CS designed the overall study. ViS, MC and PM defined the motor task. CS and GB selected the subjects and conducted all clinical evaluations. EV and VaS programmed the robot, including the Robot Training procedure, conducted all experiments and analyzed the data. EV, VaS, and ViS performed the statistical analysis. ViS and CS wrote the manuscript.

All authors read and approved the manuscript.
